# Migraine with visual aura, unilateral mydriasis and Takotsubo Syndrome. Random coincidence or common pathogenesis? The precarious balance of the autonomic nervous system and the complex relationship between brain and heart: a review of the literature

**DOI:** 10.3389/fneur.2026.1886675

**Published:** 2026-07-29

**Authors:** Stefano Messina, Eleonora Colombo, Alberto Doretti, Daniela Ungaro, Stefania Corti, Nicola Ticozzi, Monica Sciacco

**Affiliations:** 1 Department of Neurology - Laboratory of Neuroscience, IRCCS Istituto Auxologico Italiano, IRCCS, Milan, Italy; 2 Department of Pathophysiology and Transplantation (DEPT), Dino Ferrari Center, University of Milan, Milan, Italy; 3 Neuromuscular and Rare Diseases Unit, Department of Neurosciences, Fondazione IRCCS Ca’ Granda Ospedale Maggiore Policlinico, Milan, Italy

**Keywords:** autonomic nervous system unbalance, hypothalamic connections, migraine, Takotsubo Syndrome, trigeminocervical complex

## Abstract

**Objective:**

The recent examination of a woman who presented a migraine attack with visual aura and unilateral mydriasis, followed, a few days later, by an episode of Takotsubo Syndrome, prompted us to review the existing literature correlating migraine and TS as well as the most recent studies regarding the association between Takotsubo Syndrome and neurological dysfunction. Our aim is to define the role of dysautonomia and the contribution of the Autonomic Nervous System in the pathogenesis of both diseases, also, to define the functional role of the hypothalamus in the both the determination of headache attacks and the onset of clinical manifestations in other organs and tissues.

**Background:**

Migraine with aura and Takotsubo Syndrome (TS) share emotional, psychological and physical triggering factors which are implicated in the pathogenetic process. The autonomic nervous system plays a crucial, though antipodal, role in both pathologies. Indeed, migraine clinical manifestations imply the activation of the parasympathetic system whereas a massive catecholamine release following sympathetic nervous system activation is pivotal in triggering the Takotsubo Syndrome.

**Methods:**

Review of anatomy, physiology and neural regulation in both physiological and stress responses. Examination of the functional connections of the hypothalamus.

**Results and discussion:**

The coexistence of psycho-emotional stressors, endocrine/hormonal factors and a possible genetic predisposition might explain the pathophysiological overlap between these two distinct clinical manifestations. The hypothalamus is functionally connected with all the pain-related brain regions and pathways and can be responsible for the clinical manifestations in both the nervous system and the cardiovascular apparatus. Migraine is the most frequent chronic, early onset condition associated with TS.

## Introduction

Migraine is a disease of the central nervous system also characterized by clinical manifestations secondary to the activation of the peripheral nervous and vascular systems. The pathogenesis of migraine is extremely complex and, for this reason, not yet fully understood. Indeed, the pain component, although representing the most consistent aspect of this pathology, is part of a rich and differentiated symptomatic process which sets in in consecutive temporal phases and which is the result of the activation of cortical and subcortical nervous/brain structures strictly interconnected by multiple neurotransmitter and neuroendocrine systems (1).

A basic role in the pathogenesis of the migraine attack is played by the trigeminovascular system which operates as a “checkpoint” for sensitive and painful stimuli eliciting from intra- and extracranial anatomical structures. Both peripheral and central nerve fibers originate from the trigeminal ganglion neurons. The former collects and transmit the sensory-painful stimuli from the meninges and intra- and extra-cranial vessels, the latter contact the nucleus of the Trigeminocervical Complex (TCC) in the brainstem. Numerous descending nerve fibers from different cortical areas, hypothalamus and brain stem nuclei, all of them regulating and modulating sensory and pain information, also converge on TCC (1, 2). Nerve fibers from the TCC projects to the superior pontine salivatory nucleus from which, in turn, parasympathetic fibers reach the sphenopalatine ganglion responsible for lacrimation and cranial arterial vasodilation (trigeminal autonomic reflex arc). The activation of the trigeminal autonomic reflex contributes to pain onset and triggers migraine autonomic symptoms (3). This widespread and complicated interneuronal and intersystemic, cortical and subcortical connectivity, which involves nervous structures with specific and different functions, accounts for the complexity of the pathology and for the extreme heterogeneity of both exogenous and endogenous factors possibly inducing the migraine attack. A step forward in the still not fully elucidated pathogenesis of migraine has been made with the discovery of the role of the Calcitonin Gene-Related Peptide (CGRP), a vasoactive peptide which is released following the activation of TCC and is responsible for the migraine attack. This finding has been crucial for the development of new migraine treatments based on CGRP-targeting drugs (4).

Takotsubo syndrome (TS) (otherwise called “broken-heart” syndrome or stress cardiomyopathy) is characterized by a transient dysfunction of the left ventricle wall, usually of the lower part (apex), resulting in a reduction in the systolic contractile capacity of the myocardium. Due to the shape of the dysfunctional apex, the syndrome is also known as “apical ballooning syndrome.” It mainly affects post-menopausal women possibly as a consequence of the increased sympathetic activation in healthy, aging women (5). The pathogenesis is not well known, but the most accredited hypothesis is that, given a favorable genetic predisposition, stressful situations, emotional and/or physical, induce an anomalous activation of the limbic system and the hypothalamic axis, determining a massive release of catecholamines in the organism with consequent damage to the microcirculation and ventricular function (5, 6).

Also, it is important to point out that, differently from what happens with migraine, CGRP is considered to have a cardioprotective effect in conditions such as heart failure and ischaemia due to its vasodilator effect. This is why migraine treatments targeting either CGRP or its receptor may have cardiovascular adverse effects (4, 7).

## Case report

The patient provided written consent for the publication of this report. The proband is a 60-year-old woman, medical doctor by profession, with healthy lifestyle habits (long walks, healthy food intake, no smoke or alcohol/drug abuse, BMI 19.7), but leading a quite stressful life, often with sleep deprivation, due to her necessity to balance job rhythms in a big metropolitan hospital and family life. Family history is negative for cardiovascular diseases, her father suffered from migraine. The patient presented two episodes of migraine without aura throughout her life, also, she suffers from rare episodes of mild headache usually located in the right lower periorbital region. The pain usually lasts two to three days, seldom requiring analgesic therapy (NSAIDs). Her past medical history is remarkable for an emergency right hemicolectomy in 2017 (Intestinal volvulus) and a Takotsubo Syndrome, triggered by a very stressful familial emotional event, in 2019. She completely recovered in both cases and she does not take any chronic home therapy.

On June 11th, 2023, in the evening, she noticed the appearance of a “black veil” in the upper quadrant of the right eye which lasted approximately 60 min in the absence of other accompanying symptoms. The following day, on awakening, the visual disturbance reappeared with the same characteristics associated with a marked and evident right eye mydriasis with normal light pupillary reflex. The neurological examination as well as a thorough neuroradiologic and ophthalmologic examination were normal which made the event consistent with a diagnosis of Benign Episodic Unilateral Mydriasis (BEUM). The mydriasis completely resolved within 24 h.

The following day, the patient experienced one of her mild cephalalgic episodes in the right periorbital area, no different from her typical ones if not for the association with a perception of “watery” right eye. She took NSAID with little or no benefit and after three days the symptoms subsided as usual.

On June 23rd, 12 days after the onset of the visual symptoms and eight days after the headache episode was over, the patient, while acting in an amateur, but demanding, theater performance, suddenly experienced exceptional / extremely high heat in her upper body, followed by intense pain in the throat and sternum as well as in the muscles of both arms. She fainted after a few seconds (bystanders refer a sudden fall forward) without any seizure-related signs and symptoms. The patient, who does not remember falling, immediately regained consciousness and was able to complete the performance even if, soon after finishing, she developed a severe orthostatic hypotension and was unable to stand without support. Chest and muscle pain had already subsided by the time she had regained consciousness.

She was taken to the Emergency Room where an echocardiography showed a dysfunction of the cardiac apex with an ejection fraction of 30%, which was consistent with a relapse of Takotsubo Syndrome. She was admitted to the Coronary Unit and she underwent a coronary angiography which was negative, thus confirming the diagnosis of stress cardiomyopathy. She gradually recovered and was dismissed after six days with an ejection fraction of 59%. She was considered fully recovered in one month.

Soon after she was discharged she confessed her family and friends that she had lived through an intense emotional pressure, also inducing sleep deprivation, in the weeks preceding the performance because of its higher quality level (new company, bigger theater) compared to what she had done in the previous years.

## Discussion

Migraine with aura and Takotsubo Syndrome are two pathologies sharing emotional, psychological and physical triggering factors, but which remain distinct in terms of pathogenetic process. This dilemma is well exemplified by the peculiar case of a woman who experienced both diseases in a short interval of time. Noteworthy, she had already suffered from TS four years before.

Migraine is a central nervous system disease involving also the peripheral and autonomic nervous system. Cortical hyperexcitability and altered neuronal functional connectivity determine an abnormal processing of exogenous and endogenous stimuli, an unbalanced activation of inhibitory and excitatory cortical and subcortical nervous structures, and, ultimately, the migraine attack. Many factors, including sleep deprivation, fasting, bright light and psychophysical stress, modify the prefrontal cortex excitability – an important center regulating cognition, behavior and emotion—and induce a dysregulation of the complex network made by cortical, subcortical and brain stem nuclei including TCC (1).

Takotsubo Syndrome is a transient dysfunction of the left ventricle wall, almost always the apex, which ultimately reduces the systolic contractile capacity of the myocardium. Even if coronary arteries are unaffected, symptoms may mimic an acute, myocardial infarction and the disease can be life threatening if unrecognized or untreated. The most accredited.

hypothesis is that, given a favorable genetic predisposition, stressful situations, emotional and/or physical, induce an anomalous activation of the limbic system and the hypothalamic axis, determining a massive release of catecholamines in the organism with consequent damage to the microcirculation and ventricular function (8–10).

To our knowledge, only few isolated cases correlating migraine and TS have been reported. In 2012, Jalan et al. (11) reported the case of a 25 y.o. female who experienced a longer than usual and particularly severe throbbing headache culminating, after 4 days of persistent pain, in cardiac manifestations – chest pain and shortness of breath – consistent with a diagnosis of TS. Given the lack of any emotional stressors prior to the event, the authors suggest that the distress caused by the status migrainosus favored a causal relationship between headache and TS as a consequence of Autonomic nervous system (ANS) imbalance.

Another case was reported by Großkopf et al. (12). The proband, a healthy 48-year-old woman, experienced her first attack of severe throbbing headache with migraine features and, when clinically evaluated, a diagnosis of TS was made based on the cardiological findings. Interestingly, she had had no thoracic symptoms, but she had experienced a psychological, family-related, stress a few days before the event. The authors suggest either the possibility that migraine somehow precipitated TS or, conversely that migraine attack was triggered by TS based on the consideration that the emotional stress can be a precursor of both events.

More recently, Desmeules and Côté (13) reported the case of a 41 y.o. woman, suffering from migraine, who experienced mild pain in the right arm, neck and back along with chest discomfort during a unilateral, severe migraine attack. A diagnosis of TS was made and the authors conclude that migraine could induce TS directly and/or following vascular dysfunction. It is peculiar to note that this patient referred the occurrence of several similar episodes over the previous three years, unfortunately she had never sought medical attention.

It is important to note that this patient was not on anti-CGRP medications, nor were the other two reported patients whose clinical event occurred before anti-CGRP treatments became available.

The contribution of the ANS to the pathogenesis of both diseases is widely recognized, most recently and thoroughly so for the clinical manifestations of headache to the point that autonomic factors currently play a major role in the development of innovative migraine treatment protocols (14, 15).

The parasympathetic and sympathetic nervous systems – the two division of the ANS—act independently, but their balanced interaction is required to maintain proper function of the organs and tissues they innervate (16). Dysautonomia results from an imbalance between the two systems, which can be highly dysfunctional for individuals if we consider that, besides controlling a number of physiologic functions, the ANS is also involved in the regulation of psycho-emotional behaviors and cognitive performances (17).

Considering the main clinical manifestations associated with migraine attacks – orthostatic hypotension, diarrhea, nasal congestion, lacrimation, ptosis, miosis among the most common – the imbalance seems to result from parasympathetic hyperfunction as a consequence of either increased parasympathetic or decreased sympathetic tone. The ultimate and more debilitating manifestation, pain, is also related to the dominance of the parasympathetic tone as a result of the complex interaction among different central, peripheral and autonomic pathways, including neurogenic inflammation, converging on, and thus activating, the nociceptors within the trigeminovascular system (2, 3).

However, recognized migraine-triggering external factors, among them sleep deprivation, are known to decrease parasympathetic tone and, particularly, to increase sympathetic tone (18) which brings back to the pathogenesis of Takotsubo syndrome. In addition, mydriasis results from the hypothalamic activation of the sympathetic medullary center spinal cilium C8-T1, pupillary dilatation being induced by post-ganglionic sympathetic fibers.

It has indeed been proposed that migraine could be triggered by a prolonged, stress-correlated, stimulation of the sympathetic nervous system followed by a decline in norepinephrine release and a concomitant release of neurotransmitters like dopamine and adenosine which induce vasodilatation (19).

The intriguing, apparently paradoxical, interaction between parasympathetic and sympathetic nervous system in triggering migraine attacks has been recently approached in a review by Tana et al. who specifically investigated the connection between migraine and cardiovascular disease (CVD). The authors focused their attention on the pathophysiological mechanisms, namely endothelial dysfunction, platelet activation, inflammation and autonomic imbalance, shared by the two disorders. Specifically, psychosocial stressors like anxiety disorders, which affect more than 50% of migraine patients, are associated with increased sympathetic activation, tachycardia and blood pressure variability, all of which elevate CVD risk. They therefore suggest that stress-mediated autonomic dysfunction may trigger cardiovascular manifestations in migraine subjects (20).

In this regard, a recent metanalysis by von Känel et al. suggests that TS patients show a distinct pattern of sympathetic nervous system activation, characterized by significantly elevated post- stress plasma norepinephine levels which may play a crucial role in TS pathophysiology. More specifically, under laboratory-induced stress conditions, TS-diagnosed patients showed a more pronounced, statistically significant increase in plasma norepinephrine levels than non-cardiovascular controls, despite norepinephrine plasma levels being not significantly different at baseline conditions (6). This is a quite valuable finding, even more so in our case since the patient suffers from headache and had already experienced a stress-related TS event in the past. Given her chronically stressful lifestyle, further exacerbated by the emotional stress preceding the theatrical performance, it can be argued that she was experiencing a prolonged state of severe autonomic dysregulation, a sort of “dysautonomic storm,” whose most probable early manifestation was the sympathetic-induced unilateral mydriasis rapidly evolving to migraine which finally led to TS.

Interestingly, functional, MRI-based, connectivity studies performed over the last ten years have demonstrated that hypothalamic connectivity changes are a main feature in migraine manifestations. More specifically, functional connections have been demonstrated between hypothalamus and autonomic nervous system structures in migraine patients during interictal period (21). Also, evidence has been provided that, immediately prior to a migraine headache phase, functional connectivity between lateral hypothalamus and brain regions involved in the pain-related pathways decreases (22). A thorough investigation on the involvement of the hypothalamus in the pathophysiology of headache attacks, including migraine, proposes that the hypothalamus is not only anatomically, but also functionally connected with all the pain-related brain regions and pathways, that it integrates and modulates all the sensory, physiological and cognitive signals that mediate autonomic responses, and that, ultimately, the inputs it receives right before a migraine attack alter its proper functional connectivity thus facilitating the generation of the migraine attack (23).

Thus, the hypothalamus represents the connection between brain areas on one side and TCC and autonomic nervous system on the other and any change in its physiological connectivity status is responsible for clinical manifestations in both the nervous system and in other organs and tissues including the cardiovascular apparatus.

The case we report well confirms and helps elucidate this relationship. Indeed, the clinical manifestations in our patients started with a unilateral mydriasis during migraine aura—a temporarily unusual sign of activation of the sympathetic system-continued with the onset of a usual migraine attack, and ultimately peaked with a “second wind,” this time massive release of catecholamines with consequent heart failure.

We hypothesize that our patient, who routinely experienced a physiologically stressful life rhythm, went through an additional and persistent, psycho-emotional tension, progressively increasing in view of the upcoming theatrical performance, which triggered an intense disruption of the physiological connections between brain areas and autonomic nervous system; as a result, the latter went through an imbalance which acted like a “storm” causing an unpredictable alternation of sympathetic and parasympathetic manifestations.

In line with this hypothesis, fear of speech is listed among the primary (i.e., emotional) causes of Takotsubo Syndrome (10).

Also, an aggravating condition was represented by the fact that the patient had already experienced Takotsubo Syndrome four years before following a serious familial problem (illness of a close person also listed among the primary TS causes) (10). In this regard, baseline autonomic dysfunction has been reported in some TS individuals. More specifically, some women with a TS history show catecholamine hyperreactivity and suppressed.

parasympathetic modulation of the heart in response to emotional and physical stress compared with healthy age-matched controls, a condition which persists years after the TS episode, creates a chronic ANS imbalance and makes them vulnerable to acute sympathetic triggers (9).

Besides, even if more evidence is necessary, some reports have raised the hypothesis that there may be genetic determinants of abnormal adrenergic signaling making some individuals more susceptible to TS (9).

Undoubtedly, considering what we said so far, a never-ending bond seems to exist between brain and heart and this has raised the question as of whether the presence of acute or chronic neurological conditions may place individuals at higher risk for TS and its consequences.

In this regard, a very interesting study has recently been published by Santoro et al. (24) in which a population of 2,301 TS patients has been examined to look for possible concomitance of neurological disorders, the aim being to identify a TS subgroup at higher risk for negative outcome. In total, 400 TS individuals, corresponding to 17% of the cases, suffered a neurological condition, the most represented being previous cerebrovascular events (39%), neurodegenerative disorders (30.7%), migraine (10%), epilepsy (9.5%) and brain tumors (5%). Patients affected with neurodegenerative disorders had the worst both short-and long-term TS outcome, whereas the TS-migraine group had the most successful prognosis. More specifically, the migraine-associated group included younger, mainly female individuals, whose TS was quite often triggered by an emotional distress and associated with fewer complications and better prognosis. Similar results had been reported in a large US registry including TS patients (25).

These data confirm that migraine is the most frequent chronic, early onset condition associated with TS, the latter being the acute, fortunately not so frequent manifestation of the ANS dysfunction triggered by the migraine-associated psycho-emotional susceptibility.

This ANS–related pathophysiological substrate favors the concept that migraine may be regarded as a multi-organ disorder. Daily life-related stressors and hormonal factors account for the prevalence in the female, sometimes post-menopausal, population.
